# Bioinformatics analysis of the antigenic epitopes of L7/L12 protein in the B- and T-cells active against Brucella melitensis

**DOI:** 10.1099/acmi.0.000786.v3

**Published:** 2024-10-11

**Authors:** Jingjie Zhang, Huricha Baigued, Shana Chen, Haiyan Borigen, Tana Tana, Fu Quan, Dezhi Yang

**Affiliations:** 1 Department of Clinical Laboratory, Affiliated Hospital of Inner Mongolia Medical University, Hohhot 010110, PR China; 2 Pharmaceutical Laboratory, Inner Mongolia International Mongolian Hospital, Hohhot 010065, PR China; 3 School of Chemistry and Chemical Engineering, Inner Mongolia University, Hohhot 010021, PR China; 4 The Inner Mongolia Autonomous Region Comprehensive Center of Disease Control and Prevention, Hohhot 010051, PR China

**Keywords:** antigenic epitopes, bioinformatics analysis, L7/L12 protein

## Abstract

Abstract

The objective is to analyse the physicochemical properties, spatial structure and protein–protein interactions (PPIs) of L7/L12 protein using bioinformatics methods and predict their B- and T-cell epitopes to lay a theoretical foundation for developing a novel multiepitope vaccine (MEV). The National Center for Biotechnology Information (NCBI) database was searched for the amino acid sequences of L7/L12 from *Brucella melitensis*. In addition, the online softwares, ProtParam and ProtScale, were used to predict the physicochemical properties: NetPhos3.1 and CD-search to predict the phosphorylation sites and conserved domains; SOMPA and SWISS-MODEL to predict the secondary and tertiary structures; the STRING database to analyse the PPIs; and the IEDB, ABCpred, SVMTrip and SYFPEITHI databases to predict the B- and T-cell epitopes. L7/L12 was docked to Toll-like receptor 4 (TLR4), B-cell receptor (BCR), Major histocompatibility complex I-T cell receptor (MHC I-TCR) and MHC II-TCR complexes, respectively, and the binding ability of L7/L12 to the targeted receptors was tested. L7/L12, consisting of 124 amino acids, was determined to be a stable, intracellular, hydrophilic protein containing 6 phosphorylation sites and ribosomal protein-related conserved domains. α-helices accounted for 70.16 %, β-turns for 2.42 %, extended strands for 8.87 % and irregular coils for 18.55 % of the secondary structure. The PPIs indicated that L7/L12 was involved in the constitution of ribosomes and regulating the accuracy of the translation process. Three B-cells, two cytotoxic T lymphocytes and three helper T lymphocyte epitopes were finally screened by comparing multiple databases. L7/L12 binds to TLR4, BCR, MHC I-TCR and MHC II-TCR complexes and forms stable hydrogen bonds, respectively. L7/L12, which governs the translation curate of proteins, possesses several potentially advantageous epitopes, laying a theoretical foundation for designing MEVs.

## Data Summary

All data are available in the article.

## Introduction

*Brucella melitensis* is a gram-negative, facultative intracellular bacterium that causes Brucellosis in animals and humans; thus, it is a zoonotic pathogen [1]. Humans can develop Brucellosis through direct or indirect contact with infected animals, their secretions and products derived from them [2]. Owing to the popularity of live, attenuated veterinary vaccines in recent years, Brucellosis has decreased globally. However, it persists in developing countries [3]. *B. melitensis* invades cells to survive and reproduce, leading to abortion and infertility in livestock and damage to multiple human organs, seriously affecting animal husbandry as well as threatening human health [4].

Vaccination is currently the most effective way to prevent and control Brucellosis. Live attenuated vaccines have been widely used in protecting livestock from diseases. Nonetheless, certain defects, such as the ability to induce abortion in pregnant animals, interference with diagnostic testing and human toxicity, are among the many concerns [5]. Therefore, developing safer and more effective *B. melitensis* vaccines for human use has become a research hotspot.

The ribosomal protein, L7/L12, with a conserved DNA sequence and a high degree of homology in most *Brucella* spp., meets the criteria for a potential vaccine candidate [6]. L7/L12 acted as an immunoprotected and dominant antigen capable of causing a delayed hypersensitive reaction in guinea pigs sensitized with *Brucella* [7,8]. L7/L12, usually in combination with other antigens of *B. melitensis*, was used to prepare a multivalent vaccine that provided a higher level of protection with a stronger immunity than live attenuated vaccines [9,10]. This observation inspired the idea of combining antigenic determinants of multiple antigens to constitute a multiepitope vaccine (MEV). MEVs demonstrated a solid and stable binding effect during the mock docking of Toll-like receptor 4 (TLR4) that can induce humoral and cellular immune responses in animal models [11]. The study analysed the structure, function and biological properties of L7/L12 by bioinformatics methods to predict its antigenic epitopes, providing a theoretical basis for preparing novel vaccine candidates for preventing Brucellosis.

## Methods

### Acquisition of the amino acid sequence

The 124 amino acid long, full-length sequences of L7/L12 from *B. melitensis* (GenBank: AAL51929.1) were retrieved from the National Center for Biotechnology Information (NCBI) database.

### Prediction of the physicochemical properties

The physicochemical properties, such as the amino acid composition, molecular mass, theoretical isoelectric point, atomic composition, instability coefficients and hydrophilicity of L7/L12, were predicted using the online software ProtParam (https://web.expasy.org/protparam/). The hydrophilicity was predicted using ProtScale (https://web.expasy.org/protparam/).

### Prediction of the phosphorylation sites and conserved domains

The phosphorylation sites and conserved domains of L7/L12 were predicted using the online softwares NetPhos3.1 (https://services.healthtech.dtu.dk/services/NetPhos-3.1/) and CD-search (https://www.ncbi.nlm.nih.gov/Structure/cdd/wrpsb.cgi), respectively.

### Spatial structure prediction

The secondary structure of L7/L12, which mainly includes the distribution, position and ratio of α-helices, β-turns and random curls, was predicted using the online tool SOMPA (https://npsa-pbil.ibcp.fr/cgi-bin/npsa_automat.pl?page=npsa_sopma.html). The tertiary structure of L7/L12 was predicted by homology modelling with the SWISS-MODEL online server (https://swissmodel.expasy.org/)https://swissmodel.expasy.org/.

### Prediction of interacting proteins

Proteins interacting with L7/L12 were predicted using the STRING database (https://cn.string-db.org/).

### Prediction of B-cell epitopes

The B-cell epitopes of L7/L12 were predicted using a combination of the online prediction software IEDB (https://www.iedb.org/https://www.iedb.org/), ABCpred (https://webs.iiitd.edu.in/raghava/abcpred/ ABC_submission.html) and SVMTrip databases (http://sysbio.unl.edu/SVMTriP/) [12]. The B-cell antigenic determinant clusters were retrieved from the IEDB database using the BepiPred Linear Epitope Prediction 2.0 method, with a response threshold >0.5 as the screening condition.

### Prediction of T-cell epitopes

T-cell epitopes of L7/L12 were predicted using a combination of the online prediction software IEDB and the SYFPEITHI database (http://www.syfpeithi.de/bin/MHCServer.dll/EpitopePrediction.htm). Human leucocyte antigen (HLA), the human major histocompatibility complex (MHC), is classified into class I and class II, which activate cytotoxic T lymphocytes (CTLs) and helper T lymphocytes (HTLs) in the human body, respectively. Based on the Chinese common and confirmed HLA allele list Common and well-documented (CWD), seven alleles were selected for MHC class I, and five alleles were selected for MHC class II molecules (Table 1). The CTL antigenic epitopes were screened as follows: peptide fragments with scores ranked in the top 1 % were selected using NetMHC pan EL 4.1 from the IEDB database, and those with affinity IC_50_ values <500 nmol/L were selected using NetMHC pan 3.0. Next, the immunogenicity was examined using MHC I Immunogenicity, with a score >0.2 as the predicted result. Those for HTLs were screened by selecting peptide fragments with the top 0.2 % percentile rankings with NetMHC II panvEL 4.0 from the IEDB database [13]. The SYFPEITHI database was applied to predict the CTL and HTL-cellular epitopes, and peptide fragments with scores >20 were selected.

**Table 1.** Selection of alleles

MHC	Locus	No.	Allele
MHC I	HLA-A	3	HLA-A*02 : 01
			HLA-A*11 : 01
			HLA-A*24 : 02
	HLA-B	2	HLA-B*40 : 01
			HLA-B*46 : 01
	HLA-C	2	HLA-C*01 : 02
			HLA-C*07:02
MHC II	HLA-DRB1	5	HLA-DRB1*03 : 01
			HLA-DRB1*07 : 01
			HLA-DRB1*09 : 01
			HLA-DRB1*12 : 02
			HLA-DRB1*15 : 01

### Prediction of allergenicity and toxicity of epitopes

The ToxinPred server (https://webs.iiitd.edu.in/raghava/toxinpred/) examined each epitope for toxicity, whereas the AllergenFP server (https://ddg-pharmfac.net/AllergenFP/) measured the allergenic properties of each epitope.

### Molecular docking

Molecular docking is based on the existing protein 3D structure and the principle of energy minimization to efficiently predict the interaction between receptors and ligands. The L7/L12 protein was downloaded from the UniPort database, and the crystal structure of the receptor protein used for docking was downloaded from the The Research Collaboratory for Structural Bioinformatics Protein Data Bank (RCSB PDB) database (https://www.rcsb.org/). MHC I and II class molecules selected the most common alleles HLA-A*02 : 01 and HLA-DRB1*15 : 01 in the Chinese population to form a complex with the corresponding T cell receptor (TCR). The L7/L12 (ID: P0A468) protein was docked with the corresponding receptors: with B-cell receptor (BCR ID: 5IFH), with TLR4 receptor (ID: 4G8A), with HLA-A*02 : 01 (ID: 6TDS) and CTL surface TCR (ID: 5YXN) and with HLA-DRB1*15 : 01 (ID: 8TBP) and HTL surface TCR (ID: 1YMM).

## Results

### Physicochemical properties of L7/L12

The physicochemical properties of L7/L12 protein were analysed by PortParam, which revealed that L7/L12 was 124 amino acids long, with a molecular weight of 12.5 KD, a theoretical isoelectric point of 4.79, a total number of 1813 atoms and a molecular formula of C_555_H_932_N_146_O_179_S_1_. In total, 17 types of amino acids were identified, the highest content of which was alanine (Ala), accounting for 24.2 % (Table 2), with 16 positively charged [arginine (Arg) or lysine (Lys)] and 22 negatively charged [aspartic acid (Asp) or glusate (Glu)] residues. The protein instability index was 18.55 (>40 is considered unstable), indicating its stability. The aliphatic index was 108.87, and the grand average of hydropathicity was 0.119, indicating that the protein was hydrophilic.

**Table 2.** L7/L12 protein amino acid composition

Amino acid	Quantity	Percent	Amino acid	Quantity	Percent
Alanine Ala	30	24.20%	Isoleucine Ile	4	3.20%
Aspartic acid Asp	6	4.80%	Methionine Met	1	0.80%
Asparagine Asn	2	1.60%	Phenylalanine Phe	1	0.80%
Arginine Arg	1	0.80%	Proline Pro	3	2.40%
Glutamic acid Glu	16	12.90%	Serine Ser	4	3.20%
Glycine Gly	10	8.10%	Threonine Thr	3	2.40%
Glutamine Gln	1	0.80%	Tryptophane Trp	1	0.80%
Lysine Lys	15	12.10%	Valine Val	112	9.70%
Leucine Leu	14	11.30%			

**Fig. 1.**
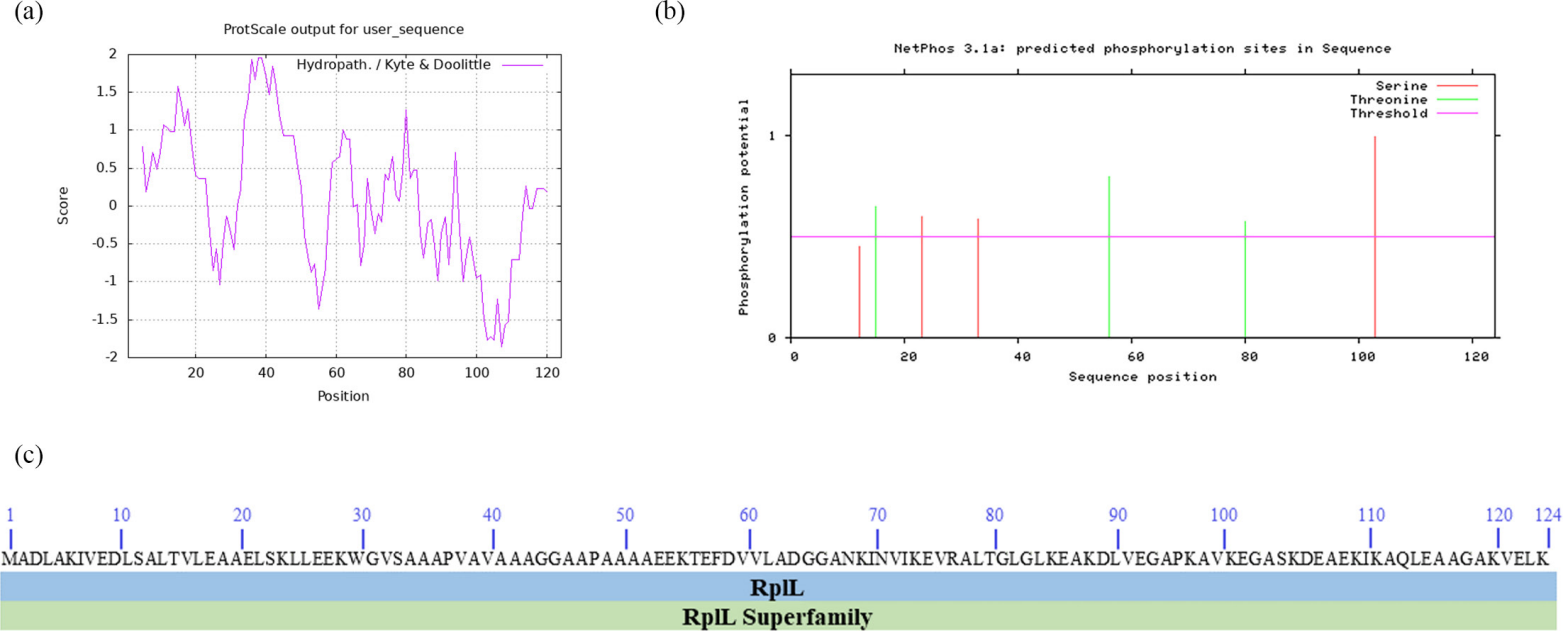
(**a**) Prediction of the hydrophilicity/hydrophobicity of L7/L12. (**b**) Phosphorylation sites in L7/L12. (**c**) Conserved domains in L7/L12.

The hydrophilicity and hydrophobicity of L7/L12 were predicted using ProtScale, which demonstrated that the protein had a maximum hydrophobicity value of 2.4 at amino acid residue 20 and a minimum hydrophilicity value of − 2.567 at 114 and 115 (Fig. 1a). The negative scores in most regions of L7/L12 indicated that the protein was hydrophilic, consistent with the predictions obtained by PortParam.

### Prediction of protein phosphorylation sites and conserved domains in L7/L12

NetPhos3.1 was used to predict the phosphorylation sites in L7/L12. At a threshold value of 0.5, the protein was predicted to possess 6 phosphorylation sites (Fig. 1b), including three Thr and Ser residues each. CD-search revealed that L7/L12 belonged to the RplL superfamily, which is involved in the composition of ribosomal structures, translation and biosynthesis (Fig. 1c).

### Prediction of the spatial structure of L7/L12

**Fig. 2.**
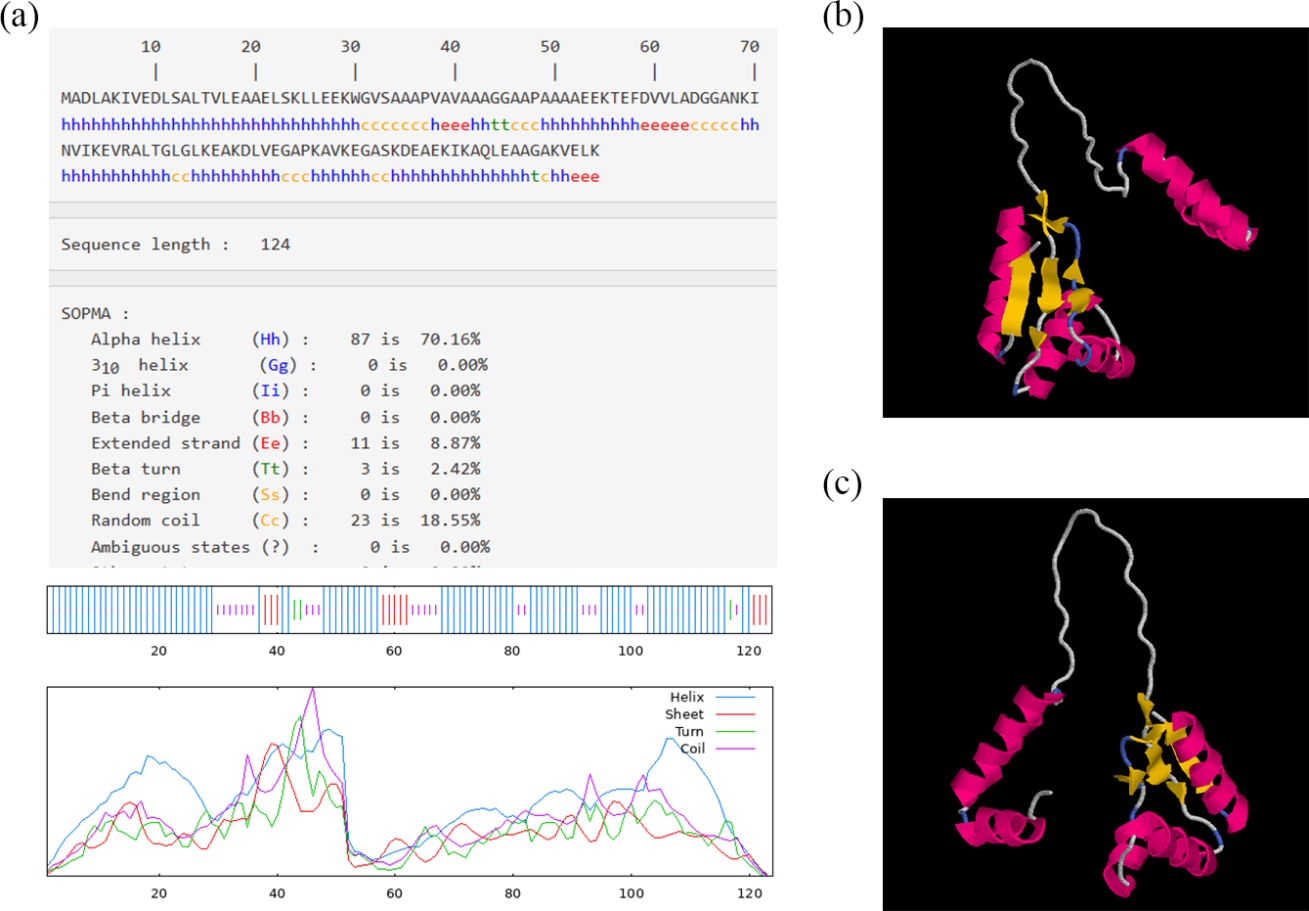
Prediction of secondary and tertiary structure of L7/L12 protein. (**a**) Prediction of the secondary structure of L7L12. (**b**) and (**c**) Homology modelling of the tertiary structure of L7/L12. The α-helices are marked in red, the β-folds in yellow, the β-turns in blue and other amino acid residues in white. (**b**) The front views of L7/L12. (**c**) The reverse pictures of L7/L12.

The secondary structure of L7/L12 was predicted by The self-optimized prediction method alignment (SOPMA), which indicated that 87 amino acids were involved in the formation of α-helices, accounting for 70.16 % of the protein; three in the formation of β-turns, accounting for 2.42 %; 11 in the formation of extended strands, accounting for 8.87 %; and 23 in the formation of irregular curls, accounting for 18.55 % (Fig. 2a). The irregular curls and β-turns are often used as candidate peptides for antigenic epitopes because their loosely arranged structures can readily change their conformations and are exposed on the protein surface to bind with antibodies.

Homology modelling of the tertiary structure of L7/L12 using SWISS-MODEL matched to the A5VR16.1, a template derived from the monomeric form of the *B. melitensis* ribosomal large subunit protein bL12. The Global Model Quality Estimation (GMQE) value was 0.78, sequence identity (Seq Identity) was 99.19 % and coverage value was 1. In addition, a visualization of this model using the software RasMol 2.7.5 (Fig. 2b, c) revealed that the β-turns and irregular curls were located near the surface of the protein. Therefore, it was hypothesized that these sites possess great potential as epitopes for use in vaccines.

### Prediction of L7/L12 protein interactions

The STRING database was used to predict the protein interaction network of L7/L12, with the ten topmost proteins interacting with L7/L12 being RpmF, RpmA, RplS, RplU, RplK, RplA, RplJ, RplC, RplD and RplB. All of these demonstrated interaction scores >0.99 with L7/L12, suggesting the high credibility of the interactions. RpmF and RpmA belong to bacterial ribosomal proteins BL32 and BL27, respectively. L7/L12, RplK and RplJ are parts of the ribosomal stalk that helps the ribosome interact with Guanosine triphosphate (GTP)-bound translation factors, which are essential for accurate translation. RplC, RplD and RplB are major rRNA-binding proteins that play a crucial role in 50S subunit assembly and tRNA binding. The RplS is localized to the 30S–50S ribosomal subunit and may play a role in the structure and function of the aminoacyl tRNA binding site. Based on the above predictive interactions, L7/L12 proteins form a part of the ribosome and interact with other ribosomal proteins to regulate the binding of ribosomes to translation factors, thus participating in the accurate translation of proteins.

### B-cell epitope analysis of L7/L12

**Fig. 3.**
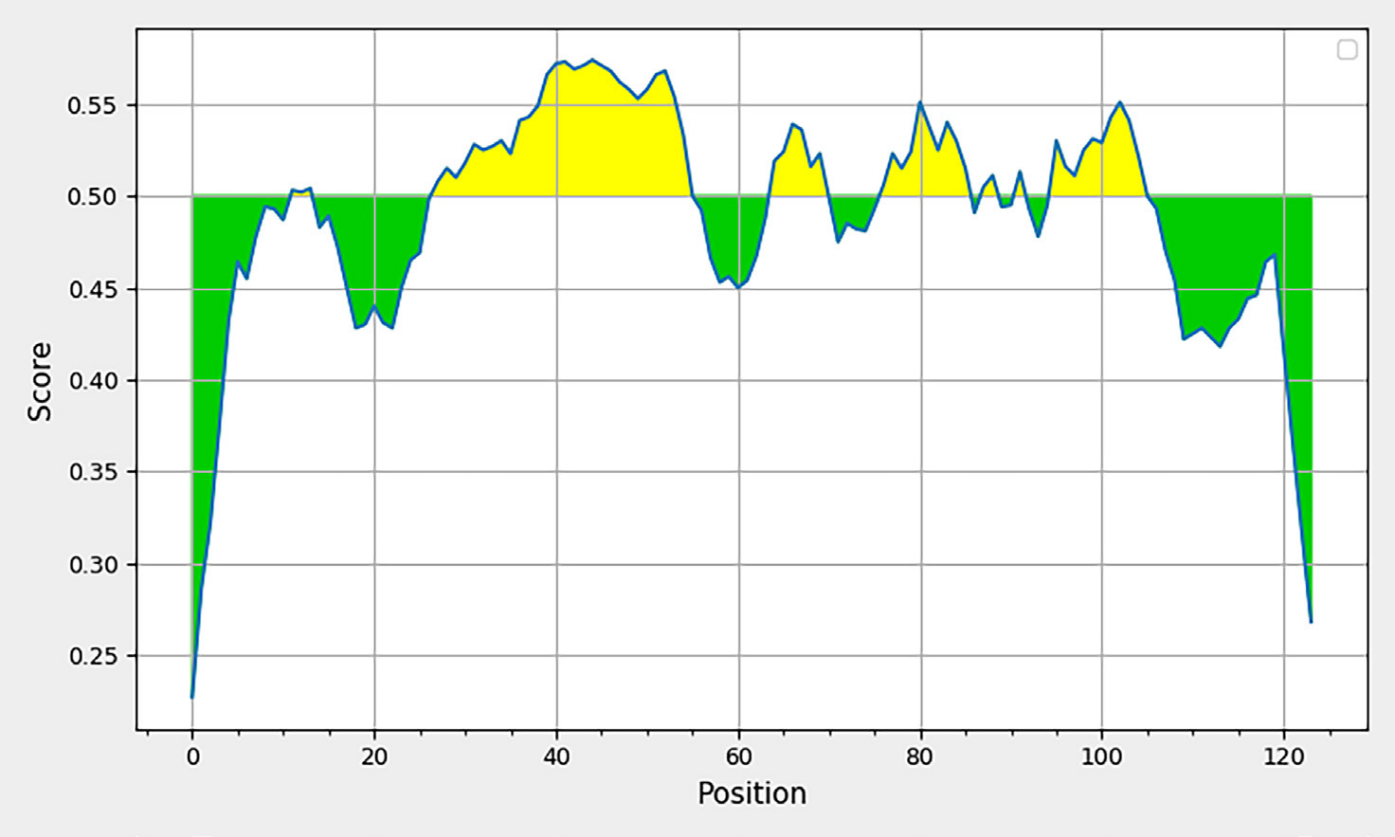
B-cell response sites of L7/L12.

B-cell responsive epitopes of L7/L12 were predicted using BepiPred in the IEDB database (Fig. 3), with four peptides having B-cell responsive epitopes for peptide chains with length ≥6 and a threshold of 0.5. The top four peptides with the highest scores were selected using ABCpred. One peptide recommended as a linear antigenic epitope was predicted using SVMTrip. Combining the BepiPred, ABCpred and SVMTrip predictive results indicated that amino acids L_44–55_ (GGAAPAAAAEEK), L_77–87_ (RALTGLGLKE) and L_97–106_ (AVKEGASKDE) can be used as the final B-cell candidate epitopes for L7/L12 (Table 3, red font with underline).

**Table 3.** B-cell epitope prediction results of L7/L12 protein

Methods	Position	Length	Sequence	Score
BepiPred	28	28	EKWGVSAAAPVAVAAAGGAAPAAAAEEK	0.6
	77	10	RALTGLGLKE	0.6
	65	6	GGANKI	0.5
	96	11	KAVKEGASKDE	0.5
ABCpred	49	16	AAAAEEKTEFDVVLAD	0.9
	88	16	KDLVEGAPKAVKEGAS	0.7
	97	16	AVKEGASKDEAEKIKA	0.7
	106	16	EAEKIKAQLEAAGAKV	0.7
SVMTrip	3	16	DLAKIVEDLSALTVLE	1.0

### T-cell epitope analysis of L7/L12

T-cell epitopes of L7/L12 were predicted using a combination of IEDB and SYFPEITHI databases. The results of the CTL epitope prediction are shown in Table 4, red font with underline, and HTL in Table 5, red font with underline. Therefore, the overlapping sequences with higher scores were selected as candidate epitopes. Finally, the amino acids L_24–32_ (KLLEEKWGV) and L_73–82_ (IKEVRALTGL) were selected as CTL epitopes, while L_27–41_ (EEKWGVSAAAPVAVA), L_58–72_ (FDVVLADGGANKINV) and L_76–87_ (VRALTGLGLKEA) as HTL epitopes of L7/L12 (summarized in Table 6).

**Table 4.** CTL epitope prediction results of L7/L12 protein

Methods	Allele	Position	Length	Sequence	Score
IEDB	HLA-A*02 : 01	24	10	KLLEEKWGVS	0.25
	HLA-A*11 : 01	46	10	AAPAAAAEEK	0.29
	HLA-A*11 : 01	11	14	LSALTVLEAAELSK	0.23
	HLA-A*11 : 01	112	9	AQLEAAGAK	0.22
	HLA-B*40 : 01	73	10	IKEVRALTGL	0.20
SYFPEITHI	HLA-A*02 : 01	6	9	KIVEDLSAL	26
	HLA-A*02 : 01	24	9	KLLEEKWGV	26
	HLA-A*02 : 01	62	9	LADGGANKI	21
	HLA-A*02 : 01	71	9	NVIKEVRAL	21
	HLA-A*02 : 01	83	9	GLKEAKDLV	22
	HLA-A*02 : 01	113	9	QLEAAGAKV	23
	HLA-A*11 : 01	16	9	VLEAAELSK	21
	HLA-B*40 : 01	27	9	EEKWGVSAA	22
	HLA-B*40 : 01	99	9	KEGASKDEA	21

**Table 5.** HTL epitope prediction results of L7/L12 protein

Methods	Allele	Position	Length	Sequence	Score
IEDB	HLA-DRB1*09 : 01	27	15	EEKWGVSAAAPVAVA	0.01
	HLA-DRB1*12 : 02	73	15	IKEVRALTGLGLKEA	0.12
SYFPEITHI	HLA-DRB1*03 : 01	4	15	LAKIVEDLSALTVLE	25
	HLA-DRB1*03 : 01	58	15	FDVVLADGGANKINV	25
	HLA-DRB1*07 : 01	27	15	EEKWGVSAAAPVAVA	24
	HLA-DRB1*07 : 01	67	15	ANKINVIKEVRALTG	24
	HLA-DRB1*07 : 01	14	15	LTVLEAAELSKLLEE	22
	HLA-DRB1*15 : 01	5	15	AKIVEDLSALTVLEA	24
	HLA-DRB1*15 : 01	23	15	SKLLEEKWGVSAAAP	24
	HLA-DRB1*15 : 01	70	15	INVIKEVRALTGLGL	24

**Table 6.** B-cell and T-cell epitope prediction results of L7/L12 protein

	Methods	Position	Sequence
B epitopes	Bepi Pred, ABC Pred, SVMTrip	44	GGAAPAAAAEEK
		77	RALTGLGLKE
		97	AVKEGASKDE
CTL epitopes	IEDB, SYFPEITHI	24	KLLEEKWGV
		73	IKEVRALTGL
HTL epitopes	IEDB, SYFPEITHI	27	EEKWGVSAAAPVAVA
		58	FDVVLADGGANKINV
		76	VRALTGLGLKEA

### Analysis of allergenicity and toxicity

Before being considered viable vaccine candidates, the expected conserved epitopes from previous stages must be analysed for allergenicity and toxicity characteristics. In the following investigation with AllergenFP and ToxinPred, we subsequently identified the non-allergic and non-toxic epitopes. Then, we found two B-cell epitopes [L_77–87_ (RALTGLGLKE) and L_97–106_ (AVKEGASKDE)], one CTL epitope [L_73–82_ (IKEVRALTGL)] and three HTL epitopes [L_27–41_ (EEKWGVSAAAPVAVA), L_58–72_ (FDVVLADGGANKINV) and L_76–87_ (VRALTGLGLKEA)] (Table 7).

**Table 7.** Allergenicity and toxicity analysis of epitopes

	Sequence	Allergenicity	Toxicity
B-cell epitopes	RALTGLGLKE	Non-allergen	Non-toxin
	AVKEGASKDE	Non-allergen	Non-toxin
CTL epitopes	IKEVRALTGL	Non-allergen	Non-toxin
HTL epitopes	EEKWGVSAAAPVAVA	Non-allergen	Non-toxin
	FDVVLADGGANKINV	Non-allergen	Non-toxin
	VRALTGLGLKEA	Non-allergen	Non-toxin

**Table 8.** L7/L12 amino acids that form hydrogen bonds with targeted receptors

L7/L12 amino acid (position)-BCR amino acid (position)	L7/L12 amino acid (position)-TLR4 amino acid (position)	L7/L12 amino acid (position)-MHC Ⅰ-TCR amino acid (position)	L7/L12 amino acid (position)-MHC Ⅱ-TCR amino acid (position)
Ser (103)-Tyr (57)	Asn (68)-Arg (382)	Thr (80)-Lys (56)	Glu (106)-Ser (58)
Glu (106)-Tyr (57)	Lys (109)-Glu (474)	Gly (81)-Tyr (99)	Glu (100)-Arg (60)
Glu (75)-Ser (31)	Asn (71)-Asp (453)	Gly (81)-Tyr (34)	Asp (59)-Arg (80)
Ala (78)-Ser (33)	Asn (71)-Gln (430)	Arg (77)-Tyr (34)	Arg (77)-Phe (109)
Thr (80)-Gln (100)	Asn (71)-Lys (477)	Lys (74)-Asp (32)	Lys (88)-Glu (106)
Arg (77)-Asp (99)	Lys (74)-Asp (502)		Lys (68)-Glu (169)
Leu (84)-Tyr (48)	Lys (85)-Glu (603)		Ala (94)-His (171)

### Molecular docking

**Fig. 4.**
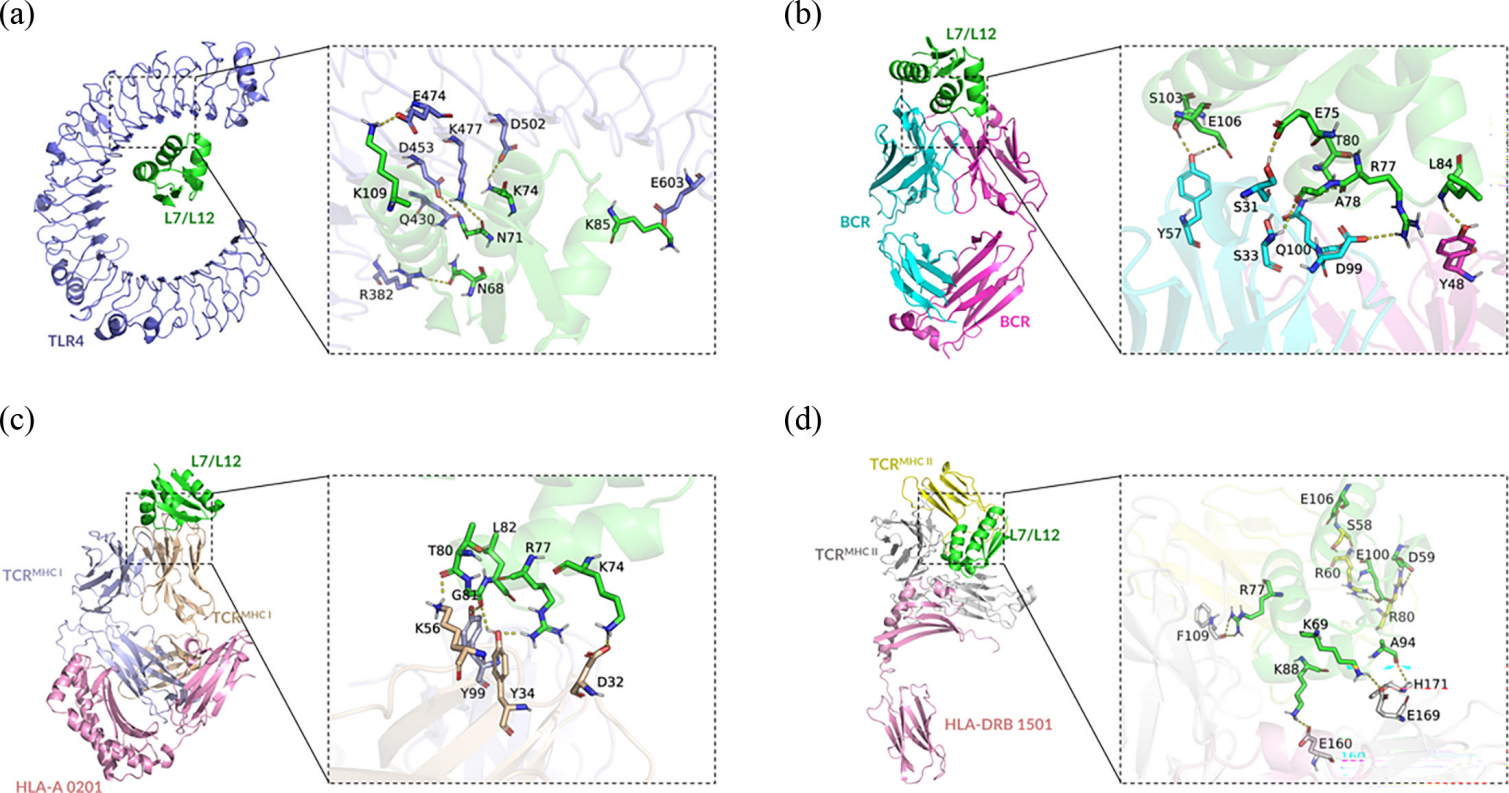
L7L12 docking with targeted receptor molecules. (**a**) Molecular docking of L7/L12 protein with TLR4. (**b**) Molecular docking of L7/L12 protein with BCR. (**c**) Molecular docking of L7/L12 protein with MHC I molecule and TCR. (**d**) Molecular docking of L7/L12 protein with MHC II molecule and TCR. The dotted lines represent hydrogen bonds.

Docking simulations showed that L7/L12 established a stable complex with the targeted receptor (Fig. 4). The binding of L7/L12 to BCR involves seven hydrogen bonds and the binding of TLR4 receptor to seven hydrogen bonds. Five hydrogen bonds are involved in the stable binding of L7/L12 and HLA-A*02 : 01-TCR complex. Seven hydrogen bonds are involved in binding to the HLA-DRB1*15 : 01-TCR complex. These hydrogen bonds and their bound amino acids are represented in Tables7  8.

## Discussions

*B. melitensis* is the most common *Brucella* species infecting humans. About half a million new cases of human infection with *B. melitensis* occur worldwide each year [14]. Following a human infection, *Brucella* species use the host’s immune system to create chronic infections. These infections can cause a variety of clinical symptoms, from arthritis and fatigue to more severe consequences like endocarditis and neurological diseases. Due to a high relapse rate of Brucellosis with single therapeutic agents, it is often treated by combinations of multiple antibiotics, mainly tetracyclines, aminoglycosides, chloramphenicol and quinolones. In addition, there is a lack of licensed vaccines against *Brucella*. Therefore, there is an urgent need for an effective vaccine-based treatment for Brucellosis in humans. As an emerging vaccine, the MEV improved safety and efficacy by linking the antigenic determinants that elicit immune responses in B- and T-cells using a bottom-up approach with reverse vaccinology and has received much attention [15,16]. MEVs prepared using the Omp10, Omp25, Omp31 and BtpB proteins of *Brucella* induced humoral and cellular immune responses in animal models [17]. Therefore, the selection of appropriate protein antigens is crucial for the design of MEVs. The ribosomal protein, L7/L12, is the dominant antigen capable of eliciting T-cell-based immunity, leading to a high degree of homology across multiple species [18,19]. In addition, the recombinant L7/L12 protein can induce Th1-type immune responses and enhance the extracellular secretion of IFN-γ in patients with acute *Brucellosis* [20]. The multiepitope vaccine candidate for *B. melitensis* has analysed some proteins, but the antigenic epitopes of the L7/L12 protein have not been reported yet [17,21]. Since L7/L12 can be used as a candidate molecule for protein vaccines [9,18, 22], it can also be used as a candidate molecule for nucleic acid vaccines. Hence, L7/L12 can be considered a potential antigen for the human *Brucella* vaccine.

Humoral immunity of an organism plays a vital role in the early stages of *Brucella* infection. Cellular immunity begins to play a crucial role when macrophages phagocytose bacteria and colonize cells through immune escape functions. CTL-mediated cell death and production of IFN-γ by helper T-cells were key mediators modulating protective immunity against infection by *Brucella* [23]. Therefore, the B-, CTL- and HTL-cellular epitopes of L7/L12 were analysed. First, IEDB, ABCpred and SVMTrip were used to predict the B-cell epitopes, with the amino acids L_44–55_ (GGAAPAAAAEEK), L_77–87_ (RALTGLGLKE) and L_97–106_ (AVKEGASKDE) identified as the final candidate B-cell epitopes for L7/L12 after selecting the overlapping sequences with high scores. Then, the IEDB and SYFPEITHI databases were applied to predict the T-cell epitopes of L7/L12. Finally, the amino acids L_24–32_ (KLLEEKWGV) and L_73–82_ (IKEVRALTGL) were selected as the CTL epitopes, while L_27–41_ (EEKWGVSAAAPVAVA), L_58–72_ (FDVVLADGGANKINV) and L_76–87_ (VRALTGLGLKEA) as HTL epitopes of L7/L12.

In the immune response against *Brucella* infections, bacterial antigens are engulfed by antigen-presenting cells and processed into antigenic epitopes [24]. These peptide fragments are presented to the cell surface and can be recognized and bound by immune cell surface receptors. We used databases such as IEDB, ABCpred and SYFPEITHI to evaluate the hydrophilicity, surface accessibility and affinity for MHC class molecules of L7/L12 protein to identify candidate epitopes that are most likely to bind to immune cell surface receptors. Molecular docking simulates the interaction between antigens and receptors, revealing their binding sites. The predicted antigen epitopes that have been identified additionally show these binding locations. For example, serine and glutamic acid amino acids in L_97-106_ can form hydrogen bonds with BCR, while tyrosine and glycine in L_73-82_ can form hydrogen bonds with MHC I-TCR complexes. The arginine of L_58-72_ can form hydrogen bonds with MHC II-TCR complexes. The antigenicity reaction measures the affinity between the proposed epitope and MHC molecules. The molecular docking score evaluated the maximum conformational stability of our proposed epitopes with MHC molecules and T-cell receptors. We chose these methods because they are highly accurate in predicting binding conformations and are more suitable for our protein–protein interaction methods.

MEVs designed based on the antigenic proteins of *Brucella* have been applied in animal models and achieved excellent immunization results [25]. The predictive analysis revealed that L7/L12 contained several potentially advantageous antigenic epitopes, especially of T-cells, which can be used as candidate antigens to prepare MEV vaccines.

## Conclusions

In the study, the physicochemical properties, structural features, protein–protein interactions and dominant epitopes of the L7/L12 ribosomal protein of *B. melitensis* were predicted using bioinformatics methods, with the potential B- and T-cell epitopes of L7/L12 identified with certainty, providing a theoretical basis for the extraction and purification of L7/L12 and the design of multiepitope vaccines for use in humans against *Brucella*.

## Abbreviations

BCR: B-cell receptor
CTL: cytotoxic T lymphocyte
CWD: common and well-documented
GMQE: Global Model Quality Estimation
GTP: guanosine triphosphate
HLA: human leucocyte antigen
HTL: helper T lymphocyte
MEV: multiepitope vaccine
MHC: major histocompatibility complex
NCBI: National Center for Biotechnology Information
PPIs: protein–protein interactions
RCSB PDB: Research Collaboratory for Structural Bioinformatics Protein Data Bank
SOPMA: self-optimized prediction method alignment
TCR: T cell receptor
TLR4: Toll-like receptor 4
